# Correction: Reusable bentonite clay: modelling and optimization of hazardous lead and *p*-nitrophenol adsorption using a response surface methodology approach

**DOI:** 10.1039/c9ra90016k

**Published:** 2019-02-28

**Authors:** Mohamed Zbair, Zakaria Anfar, Hassan Ait Ahsaine

**Affiliations:** Laboratory of Catalysis and Corrosion of Materials, Chouaïb Doukkali University, Faculty of Sciences El Jadida, BP. 20 El Jadida 24000 Morocco zbair.mohamed@gmail.com; Materials and Environment Laboratory, Ibn Zohr University, Faculty of Sciences Agadir Morocco

## Abstract

Correction for ‘Reusable bentonite clay: modelling and optimization of hazardous lead and *p*-nitrophenol adsorption using a response surface methodology approach’ by Mohamed Zbair *et al.*, *RSC Adv.*, 2019, **9**, 5756–5769.

In the published article there was an error in the naming of the pollutant Pb^2+^ in Table 9 on p. 5763. The correct version of this table is shown here below.

**Table:** Thermodynamic parameters for Pb^2+^ and *p*-nitrophenol adsorption onto BC-500

	Δ*H* (kJ mol^−1^)	Δ*S* (kJ mol^−1^ K^−1^)		Δ*G* (kJ mol^−1^)		
				293 K	313 K	333 K
Pb^2+^	−104.499	−0.201	*K*_C_	184 568 580	3 415 381.2	1 126 960.8
				−46.365	−39.148	−38.580
*p*-Nitrophenol	−132.467	−0.303	*K*_C_	123 915 710.3	362 868.435	199 963.6695
				−463.657	−33.313	−33.792

The Royal Society of Chemistry apologises for these errors and any consequent inconvenience to authors and readers.

## Supplementary Material

